# Statutory retirement and changes in self-reported leisure-time physical activity: a follow-up study with three time-points

**DOI:** 10.1186/s12889-017-4455-9

**Published:** 2017-05-30

**Authors:** Ansku Holstila, Minna Mänty, Ossi Rahkonen, Eero Lahelma, Jouni Lahti

**Affiliations:** Department of Public Health, University of Helsinki, P.O. Box 20, 00014 University of Helsinki, Helsinki, Finland

**Keywords:** Exercise, Life-course transitions, Older adults, Employment status, Longitudinal study

## Abstract

**Background:**

Retirement is a key life event, which is associated with changes in physical activity, however, there is limited evidence with regard to changes in physical activity that take place in post-retirement years. The aim of this study was to examine how leisure-time physical activity changes shortly after the transition to retirement and during the post-retirement years.

**M**ethods**:**

The phase 1 data were collected in 2000–2002 (n = 8960, response rate 67%) among 40–60-year-old employees of the City of Helsinki, Finland. Phase 2 was carried out in 2007 (*n* = 7332, response rate 83%) and phase 3 in 2012 (*n* = 6814, response rate 79%). Disability retirees and those under the age of 50 at baseline were excluded. This yielded 2902 participants. Most of the participants (79%) were women. The mean age of the participants was 54.4 in phase 1. Negative binomial models for repeated measurements with generalized estimating equations (GEE) were used to calculate the incidence rate ratios (IRR) and 95% confidence intervals (CI). These indicated the changes in time spent in self-reported leisure-time physical activity among the retired compared with the continuously employed.

**Results:**

Of the participants, 851 retired on the grounds of old age during the first period (phases 1–2), and 948 during the second period (phases 2–3). Change in physical activity was positive among those who retired during the first (IRR = 1.10, 95% CI 1.04–1.17) and second (IRR = 1.10, 95% CI 1.04–1.16) periods compared to the continuously employed. During the second period, there was little difference between those who had retired during the first one (IRR = 0.96, 95% CI 0.91–1.02) and the continuously employed.

**Conclusions:**

The transition to statutory retirement was associated with an immediate increase in leisure-time physical activity, which nevertheless diminished during post-retirement years.

## Background

Physical inactivity is common worldwide especially among people in older age groups, and activity tends to decrease as people get older. Almost 50 % of people 60 years and older are inactive in Europe [1, 2]. Inactivity and low levels of activity among older people are especially harmful from a public-health perspective. Physical activity is necessary for healthy ageing as it reduces functional limitations [3], falls, risk of dementia as well as many other non-communicable diseases.

Changes in physical activity during the life course are associated with major life events and transitions, such as graduation, getting married, becoming a parent or retiring [4]. Previous studies have shown an association between the transition to statutory retirement and an increase in leisure-time physical activity [5–9]. However, there is still little evidence on whether post-retirement changes in physical activity are temporary or persistent. One study [10] with a 13-year follow-up showed no increase in leisure-time physical activity among retirees, indicating that the positive effect of retirement observed in other studies may be short-termed and may not be maintained post-retirement. We are aware of only one study [11] which observed changes in physical activity also during the post-retirement years and indicated that the increase in physical activity after transition to retirement is temporary.

It has been shown in previous studies, that changes in physical activity are also associated with socioeconomic position [12, 13], and with divorce, separation or the death of a spouse or a partner [9]. In addition, limiting long-standing illness, a high body mass index and smoking can cause a decline in the level of physical functioning [14–17], and thus limit opportunities to engage in physical activity. These factors should be taken into consideration in examining the association between the transition to retirement and changes in physical activity [7].

The present study continues our earlier research [7], which revealed a positive association between changes in leisure-time physical activity related to the transition to retirement but with only one follow-up after retirement. Our study focuses on two questions: (1) Is transition to statutory retirement associated with similar changes in leisure-time physical activity in two consecutive follow-up periods? (2) How does leisure-time physical activity change over the post-retirement period?

## Methods

### Participants

This study is based on Helsinki Health Study (HHS) cohort data. The baseline (phase 1) surveys were sent by mail to all employees of the City of Helsinki aged 40, 45, 50, 55 and 60 in 2000, 2001 and 2002. Phase 2 and phase 3 questionnaire surveys were conducted in 2007 and 2012 respectively. There were 8960 respondents in phase 1 (response rate 68%), 7332 in phase 2 (response ate 83%) and 6814 in phase 3 (response rate 78%). Seventy per cent (*n* = 6245) of the participants took part in all three surveys. Non-response and attrition analyses showed that the data sufficiently represent the target population [18]. Eighty per cent of the participants were women, which represents the gender distribution among municipal employees in Finland [19].

We included 2902 participants in our analysis, having excluded those who were under 50 years old in phase 1 (*n* = 3702) because there were only a few retirees in this age group. We excluded those who retired on special grounds such as disability (*n* = 731) because the association between retirement and changes in physical activity is likely to differ in disability compared with statutory retirement. We also excluded those who were outside employment due to other reasons than retirement during the follow up (*n* = 102) and those with missing values in physical activity variables, retirement status or other covariates (*n* = 266).

### Retirement status

Retirement status was classified in three groups. Those who continued working during the entire follow-up time *(continuously employed)*, those who retired in *the first follow-up period* between phase 1 and phase 2 (*Retired1*) and those who retired in *the second follow up period* between phase 2 and phase 3 *(Retired2).* Because we measured leisure-time physical activity for the whole of the preceding year we considered those who retired within six months of responding to the survey still to be in employment.

### The measurement of physical activity

We used the same question to measure leisure-time physical activity (including commuting) in all three phases. The participants were asked how many hours a week, on average, they had spent on physical activity during the past year, on four grades of intensity equivalent to walking, brisk walking, running and jogging. The minutes spent on moderate to vigorous leisure-time physical activity were calculated by summing up the time spent on each intensity grade. The main analyses focused on the changes in time (minutes/week) spent on leisure-time physical activity. For descriptive purposes we calculated metabolic equivalent (MET) hours by multiplying the MET values of each intensity grade [20] and summing them up [21]. The participants were classified as low activity if their average weekly physical-activity level was less than 14 MET hours. The cut-off point of 14 MET –hours per week is based on the recommended level of physical activity, which is equivalent to approximately the energy expenditure 1000 kcal per week. This level of physical is adequate to offset several risk factors of inactivity. [22].

### Covariates

Gender, age, socioeconomic position (SEP) during phase 1 and time-variant marital status, smoking, limiting long-standing illness (LLI) and body mass index (BMI) were used as covariates. We dichotomized limiting longstanding illness (LLI) to those who reported a longstanding illness and that restricted working or other daily tasks and to those who did not. BMI was calculated by dividing the self-reported weight of the participants by their self-reported height in metres squared. Occupational socioeconomic position was classified in four groups: managers and professionals, semi-professionals, routine non-manual workers and manual workers. Marital status was classified as single, married or cohabiting, and divorced or widowed. Smoking status was divided into non-smokers and smokers based on self-reported regular smoking.

### Statistical methods

We used comparison of means and cross tabulations to describe the study variables. Negative binomial regression models for repeated measurements with generalized estimating equations (GEE) were used to calculate the incidence rate ratios (IRR) and their 95% confidence intervals (CI) for the changes in physical activity among the Retired1 and Retired2 groups compared with the continuously employed. Time in minutes per week was used as the outcome variable and the interaction between the phase and the retirement group was examined as a predictor. We adjusted the different covariates in three models. In the first model we adjusted for gender and age, in the second model also for SEP and time-variant marital status and the third for BMI and LLI, as well. There was no significant gender interaction (*p* = 0.2) and women and men were pooled for the analyses. We performed the analyses with SPSS version 22 for Windows.

## Results

Table 1 presents the distributions of the study variables. The respondents were fairly evenly distributed by retirement status: 1108 were continuously employed, 851 retired during the first period between phases 1 and 2 (Retired1) and 948 retired during the second period between phases 2 and 3 (Retired2). The mean age of the participants was 54.4 years. The continuously employed were younger than the average age, and those in the Retired1 group were the oldest. There were slightly fewer routine non-manual workers in the Retired1 group than in the other groups. The proportion of smokers was highest among the continuously employed. A lower proportion of participants among the continuously employed reported limiting longstanding illness (LLI) during phase 1, but this difference almost disappeared during the follow-up. The proportion of inactive participants decreased in the Retired1 group during the first period and increased during the second, and these changes were smaller among the continuously employed. The proportion of inactive members of the Retired2 group decreased during the first period, but there was no change during the second one. (Table 1).

**Table 1:** Description of the study variables by retirement status (*N* = 2902)

	Continuously employed *n* = 1103	Retired1^a^ *n* = 851	Retired2^b^ *n* = 948	All *n* = 2902	*p* χ^2^/Anova
Phase 1.					
Physical activity min/week mean (SD)	315.0 (206.5)	299.1 (195.8)	299.9 (192.3)	305.4 (198.9)	0.126
Low activity %	22.8	26.6	26.3	25.0	0.088
Age mean(SD)	51.0 (2.14)	58.5 (2.35)	54.9 (1.80)	54.4 (3.72)	<0.001
Women %	81.1	75.7	81.3	79.6	0.004
Socio-economic position %					0.023
Managers and professionals	35.1	38.2	37.5	36.2	
Semi-professionals	16.0	18.8	17.4	17.3	
Routine non-manuals	37.1	29.1	34.9	34.0	
Manuals	11.9	13.9	12.0	12.5	
Marital status %					0.235
Single	10.8	10.1	10.1	10.4	
Married or cohabiting	70.2	73.0	68.8	70.5	
Divorced or widowed	19.0	16.9	21.1	19.1	
Smokers %	22.3	14.0	18.6	18.6	<0.001
BMI mean (SD)	25.1 (4.0)	26.2 (4.0)	25.9 (4.3)	25.7 (4.1)	<0.001
LLI %	12.1	21.2	17.6	16.5	<0.001
Phase 2.					
Physical activity min/week mean (SD)	317.4 (200.8)	331.9 (197.6)	314.7 (198.5)	320.8 (199.2)	0.145
Low activity %	21.9	20.8	23.2	22.0	0.468
Marital status %					0.705
Single	10.5	10.6	9.7	10.3	
Married or cohabiting	66.3	68.6	67.6	67.4	
Divorced or widowed	23.2	20.8	22.7	22.3	
Smokers %	17.3	11.3	14.7	14.7	0.001
BMI mean (SD)	25.7 (4.3)	26.4 (4.2)	26.4 (4.8)	26.1 (4.4)	0.001
LLI %	24.5	25.0	28.5	29.5	0.092
Phase 3.					
Physical activity min/week mean (SD)	297.1 (202.7)	298.3 (199.8)	323.4 (197.9)	306.0 (200.6)	0.005
Low activity %	26.5	29.6	23.4	26.4	0.012
Marital status %					0.864
Single	11.7	10.6	10.7	11.0	
Married or cohabiting	63.2	64.9	65.2	64.3	
Divorced or widowed	25.1	24.6	24.2	24.6	
Smokers %	14.3	8.3	11.3	11.4	0.001
BMI mean (SD)	26.2 (4.7)	26.5 (4.5)	26.6 (4.8)	26.4 (4.7)	0.292
LLI %	27.6	29.5	27.3	28.0	0.053

^a^Retired between phase1 and phase2

^b^Retired between phase2 and phase3

**Fig. 1:**
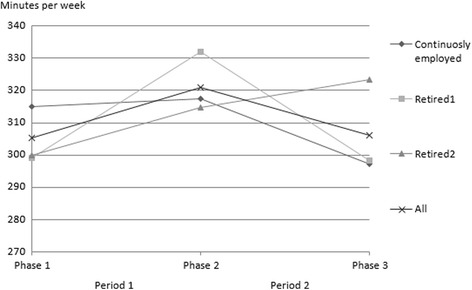
Average time spent in physical activity per week by employment status

The average amount of time spent on leisure-time physical activity in phase 1 was five hours and 15 min among the continuously employed, four hours and 59 min in the Retired1 group and five hours in the Retired2 group (Table 1; Fig. 1). During the first period the weekly amount of time being physically active increased by 30 min in the Retired1 group, changed very little among the continuously employed and increased in the Retired2 group, although less than in the Retired1 group. During the second period the average weekly time spent on leisure-time physical activity continued to increase in the Retired2 group, with a mean change of nine minutes, but decreased by 20 min and 30 min among the continuously employed and those in the Retired1 group, respectively. (Fig. 1).

During the first period, physical activity increased significantly more (IRR = 1.10, 95% CL 1.04–1.17) in the Retired1 group than among the continuously employed following adjustment for age and gender (Table 2). Further adjustments for socioeconomic position and marital status did not change the estimates, but smoking status, LLI and body-mass index attenuated the association slightly (IRR = 1.07, 95% CI 1.01–1.14). There was no difference in the change in time spent on physical activity between the Retired2 group and the continuously employed (IRR = 1.04, 95% CI 0.98–1.11). (Table 2).

**Table 2:** The association between retirement status (*N* = 2902) and change in time (min/week) spent on physical activity

	Continuously employed	Retired1		Retired2	
	IRR	IRR	95% CI	IRR	95% CI
Period 1^a^					
Model 1	1	1.10	(1.04–1.17)	1.04	(0.98–1.11)
Model 2.	1	1.10	(1.04–1.17)	1.04	(0.98–1.11)
Model 3.	1	1.07	(1.01–1.14)	1.03	(0.97–1.09)
Period 2^b^					
Model 1	1	0.96	(0.91–1.02)	1.10	(1.04–1.16)
Model 2.	1	0.96	(0.91–1.02)	1.10	(1.04–1.16)
Model 3.	1	0.95	(0.89–1.00)	1.09	(1.02–1.15)

Model 1. Adjusted for age and gender

Model 2. Model 1. + SEP and marital status

Model 3. Model 2. + smoking, LLI and BMI

^a^Period 1 is the time-period between phase 1 and 2

^b^Period 2 is the time-period between phase 2 and 3

The increase in physical activity in the Retired2 group during the second period was not as notable as in the Retired1 group during the first period. There was also a decrease in physical activity among the continuously employed during the second period, however. Thus, the differences between the retired group and the continuously employed were similar during both periods (Table1 & Fig. 1). Moreover, the age- and gender-adjusted relative difference in the changes in physical activity (IRR = 1.10, 95% CI 1.04–1.16) between the Retired2 group and the continuously employed was very similar during the second period to the difference between the Retired1 group and the continuously employed during the first period (Table 2). Adjustments for socioeconomic position and marital status did not change the associations. Adjusting for LLI and body-mass index again attenuated the association slightly (IRR = 1.08, 95% CI 1.02–1.14). The Retired1 group reduced the time spent on physical activity more than the continuously employed during the second period (Fig. 1), but the difference was not statistically significant (IRR = 0.96, 95% CI 0.91–1.02). (Table 2).

## Discussion

Our results indicate a modest increase in leisure-time physical activity after the transition to statutory retirement, but this increase does not necessarily persist over a longer period. Retirement led to an immediate positive change in physical activity in both retirement groups compared to the continuously employed, although the increases in time were only 15 and 33 min. The increase in physical activity at the time of retirement was larger in absolute numbers in the Retired1 than in the Retired2 group, but the relative difference from the continuously employed was similar. This similarity implies that the association between the transition to retirement and physical activity reported in our earlier study [7] was not attributable to a cohort effect, thus the increase in activity after retirement is not likely to relate to something that only affected employees in the Retired1 group. These results are consistent with findings from a review [6] indicating that the transition to retirement increases leisure-time physical activity.

The increase in leisure-time physical activity associated with the transition to retirement does not seem to persist. The higher activity level achieved after retirement reported in the present study was not maintained in that the time spent on leisure-time physical activity decreased among the Retired1 group over the years following retirement. However, members of this group maintained a similar level of activity as those in the continuously employed group, hence the increase in physical activity associated with the transition to retirement might partly even out the decrease associated with age. This is new evidence with regard to the association between the transition to retirement and leisure-time physical activity: several previous studies [5, 7, 8] measured physical activity only once post-retirement and did not examine the changes during the following years. Our findings indicating changes during the post-retirement period are in accordance with the results of an earlier study [10] in which no association was found between the transition to retirement and changes in leisure-time physical activity over a long 13-year follow-up and another study [11] which found an increase in physical activity during the transition to retirement that even out during the post-retirement years.

One study [23] examined psychological well-being - before and after transition to retirement and found that recently retired men felt better about their ageing process than those who had been retired for longer than two years or those who were not yet retired. These findings imply that the phase shortly after transition to retirement may be psychosocially different from later phases during the post-retirement period, this maybe one reason why the increase in physical activity after transition to retirement is short - term.

The average level of leisure-time physical activity among the participants was high at around 300 min per week during phase 1, thus the changes in time spent on such activity were quite modest in both absolute and relative terms. The changes in time spent in LTPA reported in other studies have varied. One study reported 2 h [8] increase and another around 30 min increase in physical activity [11]. In the present study the changes in the proportion of participants with low activity level (<14 MET-hours per week) were similar to the changes in time spent on physical activity. This implies that the increase in physical activity does not apply only to those who were highly active from the beginning, thus the changes are encouraging from the public-health perspective. Given the large proportion of retirees with low physical activity level in the older age group [1] and the fact that inactivity is one of the main risk factors for non-communicable diseases and disability [24], it would be ideal if the employees with low activity could become more active after the transition to retirement. However, an increase in physical activity is also likely to be beneficial to those who are already very active given the accumulating health benefits from engaging in up to five times the minimum recommended amount [25].

We focused only on leisure-time physical activity in this study. As work-related physical activity stops after retirement the total amount of physical activity may also decrease. One study [10] reported a decrease in overall physical activity after the transition to retirement, and another found an increase in total physical activity among people with sedentary jobs and a decrease among those with physically demanding jobs [26]. In addition, it should be noted that we examined the total time spent in leisure-time physical activity and separate analyses of moderate and vigorous activity was not included. Our previous study [7] showed that the level of vigorous activity was low among retirees and that the retirees increased moderate-intensity physical activity. However, it should be noted that for an older retiree the relative intensity of brisk walking may be as intense as jogging for a midlife employee. Thus it is important to examine the total amount of leisure-time physical activity.

### Strengths and limitations

Our question on physical activity included commuting physical activity, and the probable decrease in commuting physical activity after retirement is a potential cause of bias in our results. However, our findings are more likely to be conservative compared to studies that do not incorporate commuting activity, the loss of which after retirement counteracts the increases in leisure-time physical activity. Another limitation is that, we lack measurement of work related physical activity which is lost after retirement. Second, we used self-reported data on employment status and physical activity. It has been shown that self-reported measurements of physical activity may be overestimated. The questionnaire we used has not been validated, but similar questions have proved to be valid and reliable in comparison with a detailed interview [27]. Moreover, no other self-report questionnaire on physical activity has proved paramount [28]. Third, the age differences between the retirement status groups could have biased our results, given that age is associated with a decline in physical activity. We adjusted for age in our models but it had no effect on the associations. However, the age effect is likely to make our results more conservative because the increase in physical activity after retirement occurred among retirees, who were older than the continuously employed reference group used for comparison. Fourth, the cohort represent only municipal employees of the City of Helsinki which limits the generalisability of our findings.

The strengths of the study include the extensive, longitudinal, prospective dataset, and the fact that all the participants were employed and able to work during phase 1. The longitudinal data collected at three time points enabled us to extend the evidence from previous studies by examining the changes in physical activity in post-retirement years as well as the change after the transition to retirement during the two consecutive follow-up periods.

## Conclusions

Statutory retirement was associated with a short-term increase in leisure-time physical activity. However, the increase in leisure-time physical activity associated with retirement may slow down or delay the decrease in physical activity seen as people age. The results highlight the need not only to encourage people to increase their physical activity after retirement, but also to help retirees to maintain their physical activity levels in the longer term.

## Abbreviations

BMI: Body mass index
CI: Confidence interval
GEE: Generalized estimating equations
IRR: Incidence rate ratio
LLI: Limiting longstanding illness
MET: Metabolic equivalent
SEP: Socioeconomic position
